# Insecticide resistance in the field populations of the Asian tiger mosquito *Aedes albopictus* in Beijing: resistance status and associated detoxification genes

**DOI:** 10.3389/fphys.2024.1498313

**Published:** 2024-12-18

**Authors:** Xiaojie Zhou, Jing Li, Ruoyao Ni, Xinghui Qiu, Yong Zhang, Ying Tong

**Affiliations:** ^1^ Institute of Disinfection and Pest Control, Beijing Center for Disease Prevention and Control, Beijing, China; ^2^ State Key Laboratory of Integrated Management of Pest Insects and Rodents, Institute of Zoology, Chinese Academy of Sciences, Beijing, China

**Keywords:** transcriptome, insecticide resistance, cytochrome P450, metabolic resistance, *Aedes albopictus*

## Abstract

**Background:**

*Aedes (Stegomyia) albopictus* (Skuse) is an invasive and widespread mosquito species that can transmit dengue, chikungunya, yellow fever, and Zika viruses. Its control heavily relies on the use of insecticides. However, the efficacy of the insecticide-based intervention is threatened by the increasing development of resistance to available insecticides. Understanding the current status and potential mechanisms of insecticide resistance is an important prerequisite for devising strategies to maintain the sustainability of vector control programs. In this study, we investigated the current status and probable candidate detoxification genes associated with insecticide resistance in the Asian tiger mosquito in Beijing, the capital city of China.

**Methods:**

Bioassays were conducted on three field populations of *Ae. albopictus* collected from urban communities in Beijing by exposure to diagnostic doses of permethrin, deltamethrin, malathion, and propoxur. Differentially expressed genes (DEGs) associated with insecticide resistance were screened by transcriptomic analysis using Illumina RNA sequencing data (RNA-seq) from 12 independent RNA libraries constructed from female strains of the three field populations and one susceptible strain.

**Results:**

The bioassay results indicated that all the three field populations were resistant to propoxur (carbamate), deltamethrin, and permethrin (pyrethroids), but susceptible to malathion (organophosphate). Eighteen (18) cytochrome P450s (P450s), five (5) glutathione S-transferases (GSTs), four (4) carboxy/cholinesterases (CCEs), eight (8) UDP-glycosyltransferases (UGTs), and three (3) ATP-binding cassette transporters (ABCs) were found to be significantly overexpressed in the three field populations relative to the susceptible strain via transcriptomic analysis.

**Conclusion:**

This study demonstrates that the *Ae. albopictus* field populations in Beijing exhibit multiple phenotypic resistance to commonly used pyrethroids and carbamate. The identification of a number of DEGs associated with insecticide resistance indicates that the mechanisms underlying resistance in field populations are complicated, and detoxifying enzymes may play important roles. The multiple resistance status detected in the three field populations suggests that resistance management strategies such as insecticide rotation and non-chemical-based measures should be implemented in order to sustain effective control of the disease vector and vector-borne diseases.

## 1 Introduction

The Asian tiger mosquito *Aedes albopictus* (Skuse) is a highly invasive disease vector. It has been documented that this vector is able to transmit dengue, chikungunya, yellow fever, and Zika viruses through biting humans (Paixão et al., 2018) and act as a bridge vector for zoonotic pathogens to humans due to the wide host range from primary mammalian hosts to birds, reptiles, and amphibians. With limited antiviral drugs and vaccines, control of *Aedes* vectors is the primary practice to prevent dengue, Zika, and chikungunya transmission. Chemical insecticides have been used successfully for many years to control *Aedes* vectors and vector-borne diseases, due to their high efficacy and speed of action. However, the sustainability of insecticide-based vector control faces a major obstacle: the ever-increasing resistance to the few number of insecticides registered for vector control (Brogdon and McAllister, 1998).

Resistance to insecticides in *Aedes* vectors has become a global problem. Previous studies have shown that *Aedes* mosquitoes have developed resistance to the three classes of commonly used insecticides (carbamates, organophosphates, and pyrethroids) in addition to the abandoned organochlorides worldwide (Whalon et al., 2015; Moyes et al., 2017; Dusfour et al., 2015; Dusfour et al., 2019; Zulfa et al., 2022), including in China (Liu et al., 2020; Wei et al., 2021; Li et al., 2018; Li et al., 2021).

Resistance mechanisms in *Aedes* are multifaceted, involving target site insensitivity, metabolic detoxification, and cuticular modifications, each contributing differently depending on environmental pressures and genetic factors. One well-established mechanism is target site insensitivity, particularly knockdown resistance (kdr) mutations in the voltage-gated sodium channel (VGSC). A common mutation such as V1016G reduces the effectiveness of pyrethroids, one of the most widely used insecticide classes (Kasai et al., 2014). Metabolic resistance, though less explored in *Ae. albopictus* compared to other mosquito species such as *Ae. aegypti* (Schluep and Buckner, 2021) and *Anopheles gambiae* (Gueye et al., 2020), involves the overexpression of detoxifying enzymes such as cytochrome P450 monooxygenases, glutathione S-transferases (GSTs), and carboxylesterases. These enzymes degrade or sequester insecticides before they can reach their target sites. Recent studies suggest that specific P450 genes, such as those in the CYP9J subfamily, may play a role in pyrethroid resistance in *Ae. aegypti* (Dusfour et al., 2015). Cuticular resistance, involving exoskeleton changes, reduces insecticide absorption mainly by slowing its penetration due to the upregulation of cuticular protein gene expression or altered chitin biosynthesis of chitin or hydrocarbon in *Ae. aegypti* and *An. gambiae* (Jacobs et al., 2023; Balabanidou et al., 2019). The limited knowledge of metabolic resistance, especially in terms of specific enzyme contributions, hinders our understanding of how *Ae*. *albopictus* is adapting to insecticide pressures. Further research is needed to identify the mechanisms occurring in insecticide-resistant *Ae. albopictus* populations, which is crucial for developing effective control strategies.

Beijing is the capital city of China with a population of approximately 22 million. Thanks to the Healthy Beijing Program, mosquito control campaigns have been carried out since 2016 during the rainy seasons by means of larval source management and insecticide application (residual and space sprays). As a consequence, resistance to insecticides in the target mosquitoes is gradually increasing (unpublished data). Understanding the current status and mechanisms of insecticide resistance is an important prerequisite for the subsequent development of strategies to slow the development of resistance and keep the control program sustainable. As efforts in this regard, we previously completed an extensive survey on target site mutation-mediated resistance and found that multiple mutations were present in the voltage-gated sodium channel (VGSC) gene in the *Ae. albopictus* populations in Beijing (Zhou et al., 2019a). However, information on the resistance status is limited, and other possible resistance mechanisms remain unknown in *Ae. albopictus* field populations in Beijing. In this study, we attempted to identify genes that may contribute to metabolic resistance by genome-wide transcriptome analysis on three field populations of *Ae. albopictus* sampled from urban communities of Beijing.

## 2 Materials and methods

### 2.1 Mosquito samples

Larvae of *Ae. albopictus* were collected from three sampling sites in Beijing from July to August in 2019. Heping Street community (DT) in Chaoyang District and Ganjiakou community (GJK) in Haidian District were residential communities, and mosquito collection in both communities was authorized with permission from the households. JK population mosquitoes were collected from the courtyard garden of Beijing Center for Disease Prevention and Control (Beijing CDC) in Dongcheng District, surrounded by a park, residential buildings, a school, and a stadium, with permission of the authorized representative. Surroundings (within 100 m radius) of the sampling points were thoroughly searched for possible breeding sites of *Aedes* mosquitoes. All identified artificial and natural water bodies (such as tree holes, rock pools, leaf axils, bamboo stumps, and small ponds) and containers (such as bottles, tires, cans, paper cups, barrels, foam boxes, and pots) likely to harbor mosquito larvae were visually searched thoroughly for the presence of *Aedes* mosquito larvae. *Aedes* larvae were sampled using the dipping method. At least five breeding containers were used for collection at each sample site, and approximately 300 larvae were collected overall for each site/population. The susceptible strain (SS) of *Ae. albopictus* from Beijing CDC was kept in a laboratory environment without exposure to any insecticide since 1984 and showed no tolerance to different insecticides. All samples were maintained at 25°C–27°C, 60%–80% relative humidity, and a 14:10 h light:dark photoperiod. Larvae were fed with mouse feed, and adults were provided with a 10% sucrose solution and mouse for blood meal. *Ae. albopictus* specimens were identified morphologically and confirmed molecularly by the PCR method based on the rDNA-ITS sequence (Higa et al., 2010). Insecticide tolerance bioassays and transcriptome analyses were completed within the third generation (F3) after mosquito collection.

### 2.2 Insecticide resistance bioassays

All collected mosquito larvae were reared to adult mosquitoes in the laboratory, and bioassays were completed as soon as possible. Non-blood fed 3- to 5-day-old female adults of the first to third generation (F1 to F3) were used directly for bioassays without extra insecticide pre-treatment. To prepare the insecticide-impregnated paper, the insecticide was diluted to the diagnostic concentration using a mixture of liquid paraffin and ether (1:2 v/v). Next, 2 mL of the prepared insecticide solution was applied evenly onto a 15 cm × 12 cm filter paper. After allowing the ether to evaporate completely, the impregnated paper may be stored in airtight, light-protected containers or used immediately for bioassay procedures. Adults were exposed to 0.03% deltamethrin-, 0.4% permethrin-, 0.5% malathion-, and 0.05% propoxur-impregnated filter membranes according to WHO tube bioassay protocols (WHO, 2013), and the discriminating (or diagnostic) concentrations were most frequently used for *Ae. albopictus* in China (Wu et al., 2022). Meanwhile, the susceptible strain mosquito was assayed by control filter (only with the solvent and without the active ingredient) in parallel. All the test and control filters were supplied by the Division of Vector Control, Chinese Center for Disease Control and Prevention. At least triple replicates were performed per insecticide, with no less than 30 adults per replicate. After 1-h exposure, mosquitoes were transferred to a recovery tube and maintained on 10% sucrose solution for 24 h when the mortality rates were recorded. If mortality in the control exceeded 5%, Abbott’s correction was applied. According to WHO guidelines, the resistance status of mosquito populations was scored as “susceptible” if mortality at 24 h after exposure was ≥98%, “possibly resistant” if mortality ranged between 90% and 97%, and “resistant” if mortality was ≤90% (WHO, 2013; Chang et al., 2014).

### 2.3 RNA extraction, RNA-seq library preparation, sequencing, and functional annotation

Twelve (three field populations and one susceptible strain, with three replicates/pools for each population or strain) pools of 30 unfed female adults were stored in liquid nitrogen for subsequent RNA extraction. All the mosquitoes used in subsequent transcriptomic sequencing were not challenged with insecticides. Total RNA was extracted from each pool using TRIzol reagent (Invitrogen, Carlsbad, CA, United States) according to the manufacturer’s protocol. Recombinant DNase I (Takara, Japan) was used to remove potential genomic DNA. RNA integrity was evaluated using NanoDrop 2000 (Thermo Fisher Scientific, Delaware, ME, United States). RNA quality was analyzed on an Agilent 2100 Bioanalyzer (Agilent, Palo Alto, CA, United States).

The RNA-seq libraries were constructed with 500 μg of starting total RNA with the Illumina TruSeq Stranded mRNA Sample Preparation Kit (Illumina, San Diego, CA) following the kit protocol. The resulting libraries were sequenced on an Illumina HiSeq 2500 instrument to generate strand-specific, paired-end reads of length 125 bp (HiSeq SBS Kit v4 sequencing reagents). Library construction, sequencing, and read trimming were performed at the high-throughput genomics and bioinformatic analysis platform of Majorbio (Majorbio, Shanghai, China). According to FastQC version 0.11.4, no reads were tagged as poor quality. All the reads generated in Illumina-Solexa sequencing were deposited in the NCBI Sequence Read Archive (SRA) database with BioProject accession number PRJNA1110376.

The software Fastx_toolkit_0.0.14 was used to analyze the raw transcriptome data to obtain clean data, which was further aligned with the reference genome (GCF_006496715.1) using the software HISAT2 (2.2.1). The returned SAM format files were assembled using Samtools (1.9) and StringTie (2.1.2) to obtain the whole transcriptome. The assembled transcripts were annotated against NR, SWISS-Prot, Pfam, COG, GO, and KEGG.

### 2.4 Analysis of differentially expressed genes (DEGs) and functional enrichment

Gene expression level analysis was carried out using the software RSEM (1.3.3) with the transcripts per million reads (TPM) index. Expression differences of genes among different samples were analyzed using DESeq2, where the laboratory susceptible strain (SS) was used as the control and the three field-resistant populations (DT, GJK, and JK) were used as the treatment groups. The criteria for significant differences in gene expression were set as |log2Ratio| ≥ 1 and *p*-value < 0.05, where *p*-value was corrected by false discovery rate (FDR) ≤ 0.001. If there was more than one transcript for a gene, the longest transcript was used to calculate its expression level and coverage.

KEGG functional enrichment analysis of significantly differentially expressed genes in field populations was performed by Fisher’s exact test using the bioinformatics cloud platform developed by Majorbio (https://cloud.majorbio.com/). To exclude false positives for significant enrichment of KEGG Pathway, the Benjamini–Hochberg (BH) method (Benjamini et al., 2001) was used for correction with *p*-value ≤ 0.05.

In order to screen for DEGs probably involved in metabolic resistance, the following analysis pipeline was applied. First, structural domain screening was performed among the DEGs using HMMER (3.2.1) and the Pfam database, with the PF00067 domain for cytochrome P450s, PF00043 and PF02798 domains for GSTs, the PF00135 domain for CCEs, the PF00201 domain for UGTs, and the PF00005 domain for ABCs, and a total of five metabolic resistance-related gene families were searched. Second, the retrieved genes were filtered for a number of amino acid residues based on the protein sequences obtained from ORF translation using the awk command in Linux OS, and CYP450s: 450 aa–600 aa; GSTs: 200 aa–300 aa; CCEs: 500 aa–700 aa; UGTs: 500 aa–700 aa; CCEs: 500 aa–700 aa. 700 aa; UGTs: 500 aa–700 aa; ABC-transporters: 500 aa–2,000 aa corresponding genes. Then, the genes obtained from step 2 were filtered according to their expression levels using Python scripts, and genes with TPM less than 10 in all four populations were excluded.

## 3 Results

### 3.1 Phenotypic resistance

Female strains aged 3–5 days from the three field populations (GJK, DT, and JK) and the laboratory susceptible strain (SS) were bioassayed to evaluate their susceptibility to four commonly used insecticides at discriminating concentrations. In all the controls, no knock-down and dead mosquito was observed after the 1-h exposure to solvent-treated filter membrane and 24-h normal feeding. For 0.5% malathion, possible resistance (96.85% mortality) was only detected in GJK, while other two populations exhibited a mortality of more that 98%. For 0.05% propoxur, a mortality of 74.02%, 57.85%, and 50.76% was observed in DT, GJK, and JK, respectively. The three field populations exhibited significant resistance, with the mortality ranging from 61.96% to 80.13% and from 68.32% to 86.79% after exposure to 0.4% permethrin and 0.03% deltamethrin, respectively. Overall, the highest resistance level against the tested insecticides was detected in JK, except for malathion. These results indicated that the three *Ae. albopictus* field populations were resistant to permethrin, propoxur, and deltamethrin, but remain largely susceptible to malathion (Table 1).

**TABLE 1:** Resistance status of *Ae. albopictus* populations from Beijing to four insecticides by WHO tube bioassays.

Populations	Collection site	Latitude/longitude	Progeny for bioassay	History of insecticide usage	Mortality after exposure to 0.03% deltamethrin	Mortality after exposure to 0.4% permethrin	Mortality after exposure to 0.5% malathion	Mortality after exposure to 0.05% propoxur
SS	Lab breeding susceptible strain	—	—	—	100% (156)^a^	100% (121)	100% (150)	100% (149)
GJK	Ganjiakou community, Haidian District	E116.33/N39.93	F1–F3	One time of residual spray and space spray each month during July–September using pyrethroid and carbamate insecticides each year in 2016–2019	86.79% (159)	80.13% (156)	96.85% (127)	57.85% (121)
DT	Heping Street community, Chaoyang District	E116.43/N39.97	F2–F3	Two times of residual sprays and space sprays each month during July–August using pyrethroid insecticides each year in 2016–2019	76.13% (155)	61.96% (163)	100% (130)	74.02% (127)
JK	Beijing CDC, Dongcheng District	E116.41/N39.96	F2–F3	Three times of residual sprays and space sprays each month during July–September using pyrethroid and carbamate insecticides each year in 2016–2019	68.32% (161)	67.97% (153)	98.40% (125)	50.76% (130)

^a^ The data in parentheses are the number of tested insects.

### 3.2 Transcriptomes obtained from four *Ae. albopictus* populations

Sequencing the 12 transcriptomes produced 622,916,642 paired-end raw reads in total. After analysis of base composition and sequence quality using the software Fastx_toolkit_0.0.14, 618,559,408 clean reads were obtained. The error rates were below 0.03%, and Q30 percentages were > 93% (Supplementary Material 1). The clean reads reached 83.13%–85.06% coverage when mapped to the *Ae. albopictus* reference genome (GCF_006496715.1) (Supplementary Material 2).

### 3.3 Screening of differentially expressed genes associated with insecticide resistance

**FIGURE 1:**
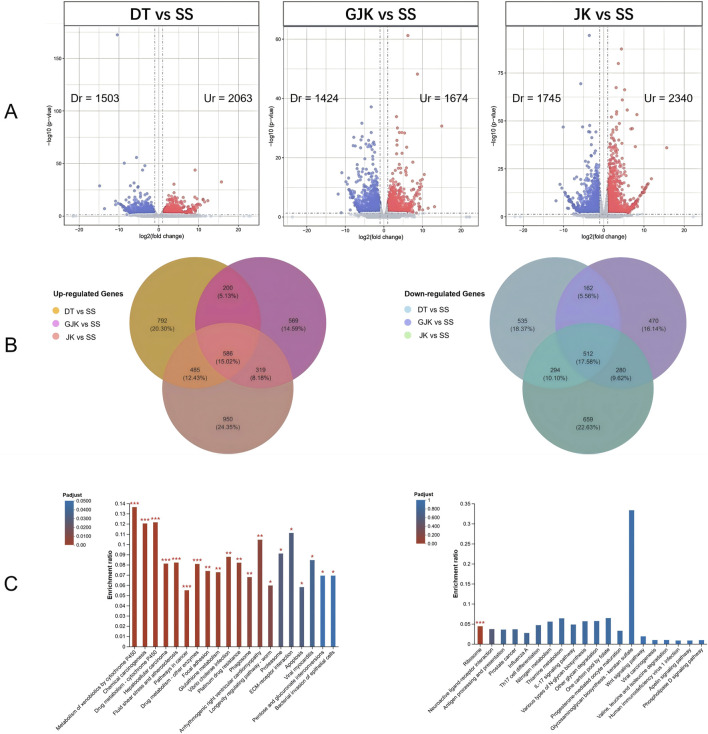
Identification of differentially expressed genes in three field populations (DT, GJK, and JK) compared to the laboratory susceptible strain. **(A)** Volcano plots (volcano plot) of differential gene expression between each of the field populations and the laboratory-sensitive populations (SS); red points represent significantly upregulated (Ur) expression genes [log2 (fold change) ≥ 1 and *p*-value ≤ 0.05], and blue points represent significantly downregulated (Dr) expression genes [log2 (fold change) ≤ −1 and *p*-value ≤ 0.05]. **(B)** Venn analysis of significantly differentially expressed genes in three field-resistant populations; the left panel shows significantly upregulated differentially expressed genes in field-resistant populations, and the right panel shows significantly downregulated differentially expressed genes in field-resistant populations. **(C)** The left side illustrates the enrichment results of genes that are commonly upregulated in three field populations, while the right side depicts the enrichment results of genes that are commonly downregulated in those same populations. The horizontal axis denotes the KEGG pathway, while the vertical axis signifies the Rich factor. The gradient of the bar colors indicates the significance of the enrichment, with a Padjust < 0.001 marked as ***, Padjust < 0.01 marked as **, and Padjust < 0.05 marked as *.

**FIGURE 2:**
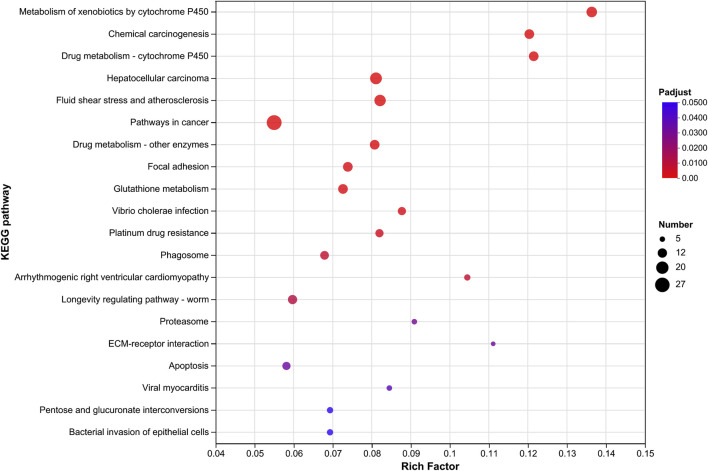
KEGG pathway enrichment analysis of significantly upregulated gene expressions in three field populations relative to the laboratory susceptible strain. The vertical axis represents the KEGG pathway, and the horizontal axis represents the Rich factor [ratio of (number of genes/transcripts enriched in the pathway) to (number of annotated genes/transcripts)]. The size of the dots indicates the number of genes in the pathway, and the color of the dots corresponds to different Padjust ranges (the smaller the Padjust, the higher the enrichment). Only the KEGG pathway with Padjust ≤ 0.05 and the top 20 enrichment are shown.

By comparing the transcriptomes, 2,063, 1,674, and 2,340 significantly upregulated (Ur) genes and 1,503, 1,424, and 1,745 significantly downregulated (Dr) genes were observed in the field-resistant DT, GJK, and JK populations, respectively, relative to the laboratory susceptible strain (SS) (Figure 1A). Considering the difference of the genetic background among the four populations, we focused on the shared subsets of genes that were differentially transcribed in all the three pairwise comparisons. In this manner, a total of 586 genes were significantly upregulated and 512 genes were significantly downregulated in the three field populations as compared with the SS strain (Figure 1B). Furthermore, KEGG pathway enrichment analyses on these commonly up- or downregulated genes in the three field populations were especially performed to further explore their potential biological functions and pathways, and the top 20 enrichment results are presented in Figure 1C. The enrichment results indicated that metabolism of xenobiotics by cytochrome P450, chemical carcinogenesis, and drug metabolism–cytochrome P450 pathways were the common significantly enriched pathways in the three field-resistant populations (Figure 2).

Focusing on xenobiotics detoxification-related genes, we found that a total of 18 P450, 5 GST, 4 CCE, 8 UGT, and 3 ABC genes were significantly overexpressed in the three field populations relative to the susceptible strain (Table 2). Among the P450s, there are 2 P450s from the CYP4 family, 6 from the CYP6 family, and 10 from the CYP9 family (9 from the CYP9J subfamily). Specifically, JK, DT, and GJK overexpressed 33, 26, and 17 detoxification-related genes, respectively. Notably, 3 P450 (CYP9J18, CYP9J32, and CYP9J74), 2 GST (GSTe2a and GSTI1), 1 CCE (COesterase6a), and 3 UGT (UGT36D1a, UGT36D1b, and UGT49C1a) genes were concurrently overexpressed in the three field-resistant populations. In addition to the above-mentioned, 8 P450s (CYP6AG17, CYP6N4, CYP6Z8, CYP6Z23, CYP9J19, CYP9J26, CYP9J65, and CYP9J68), 1 GSTs (GSTX2b), 2 CCEs (COesterase5a and COesterase6b), 3 UGTs (UGT35E2, UGT49B1, and UGT49C1b), and 3 ABCs (ABCG1, ABCG4, and ABCG1-X1) were overexpressed in two field populations.

**TABLE 2:** Xenobiotics detoxification genes that are up-regulated in the three field *Ae. Albopictus* populations relative to the susceptible strain.

Protein Family	Gene Annotation	Accession number of Source gene	Mean of TPMIn SS	DT vs SS			GJK vs SS			JK vs SS		
				Mean of TPM in DT	Fold Change	p-value	Mean of TPM in GJK	Fold Change	p-value	Mean of TPM in JK	Fold Change	p-value
P450s	*CYP4C38*	LOC109431816	10.31	33.63	3.17	8.79E-02	8.44	0.73	3.36E-01	28.51	2.22*	7.45E-12
	*CYP4D23b*	LOC109422665	4.32	6.00	1.38	5.34E-01	5.01	1.11	8.37E-01	18.20	3.68*	1.03E-09
	*CYP6AG17*	LOC115260167	12.33	58.10	4.68*	8.88E-03	24.86	1.87	1.55E-01	29.63	2.07*	1.71E-03
	*CYP6M11*	LOC109421559	19.53	62.07	3.11	1.20E-01	38.03	1.78	1.53E-01	59.73	2.45*	2.10E-10
	*CYP6N4*	LOC109409164	2.82	20.43	6.90*	4.54E-05	5.41	1.75	2.48E-01	10.80	3.14*	9.85E-07
	*CYP6N16*	LOC109398259	4.63	15.59	3.17*	4.63E-03	11.18	2.23*	4.98E-03	10.67	1.92	9.58E-04
	***CYP6Z8***	LOC109409172	31.66	184.92	5.61*	3.64E-04	66.90	1.91	5.89E-04	146.37	3.80*	7.25E-42
	***CYP6Z23***	LOC109421560	11.86	43.64	3.54*	2.65E-02	12.05	0.91	8.31E-01	31.85	2.18*	1.16E-13
	***CYP9J18***	LOC109426408	5.66	16.55	2.28*	6.26E-03	15.15	2.47*	4.25E-04	23.94	3.36*	1.20E-12
	*CYP9J19*	LOC109426410	7.72	16.26	1.84	1.04E-02	18.17	2.15*	1.05E-03	19.54	2.09*	1.98E-08
	*CYP9J26*	LOC109426416	112.61	345.79	2.93*	2.95E-02	229.39	1.87	1.10E-02	315.00	2.28*	4.83E-09
	*CYP9J32*	LOC115254008	8.48	26.87	2.77*	7.13E-06	21.11	2.25*	1.33E-02	25.19	2.39*	2.16E-07
	*CYP9J65*	LOC109403754	23.22	68.17	2.66*	3.86E-04	50.93	1.99	1.46E-04	74.68	2.64*	2.77E-20
	*CYP9J66*	LOC109403845	111.16	174.43	1.51	5.01E-01	298.24	2.50*	1.37E-03	131.71	1.00	9.92E-01
	*CYP9J68*	LOC109426407	14.37	57.61	3.60*	3.50E-06	21.82	1.38	2.26E-01	51.38	2.92*	5.14E-19
	*CYP9J72*	LOC109424185	2.37	4.86	2.13	4.26E-01	14.18	2.30*	1.09E-02	6.91	1.46	2.18E-01
	*CYP9J74*	LOC109426418	19.67	87.07	4.19*	6.00E-03	104.22	4.87*	1.17E-06	111.44	4.69*	3.73E-04
	*CYP9M6*	LOC109429650	5.33	6.84	1.13	8.37E-01	12.86	2.18*	3.41E-03	8.19	1.24	3.01E-01
GSTs	*GSTe2a*	LOC109417695	0.21	11.73	53.08*	5.84E-13	11.44	55.58*	6.60E-15	5.89	25.15*	1.47E-09
	*GSTI1*	LOC109431675	8.5	75.19	9.21*	1.67E-03	30.60	3.32*	1.66E-02	29.88	2.94*	2.80E-12
	*GSTo1*	LOC109410292	5.08	4.24	0.77	6.70E-01	11.67	2.08*	4.92E-03	12.44	1.98	9.05E-05
	*GSTT4*	LOC109412993	15.12	30.26	1.87	3.95E-01	23.05	1.33	7.53E-01	90.73	4.78*	1.64E-12
	*GSTX2b*	LOC109429681	2.22	6.14	2.68	2.43E-01	17.09	6.94*	5.59E-06	6.80	2.43*	3.20E-02
CCEs	*COesterase5a*	LOC109403315	5.22	29.31	5.29*	4.13E-04	13.90	2.38	8.68E-02	28.43	4.40*	5.18E-31
	*COesterase6a*	LOC109409692	11.37	133.11	10.52*	8.04E-12	81.64	6.33*	2.90E-10	110.31	7.78*	6.40E-30
	*COesterase6b*	LOC109414915	4.05	15.37	3.36*	5.27E-07	5.31	1.19	7.50E-01	11.95	2.40*	2.03E-08
	*Est-P*	LOC109406417	15.59	32.28	1.91	1.54E-01	25.52	1.47	1.22E-01	40.63	2.10*	8.04E-09
UGTs	*UGT2B15*	LOC109410290	5.62	11.26	1.91	2.56E-01	5.31	0.86	7.87E-01	13.78	2.03*	3.01E-05
	*UGT35E2*	LOC109424170	10.68	52.83	4.95*	1.29E-02	18.80	1.63	4.54E-01	42.7	3.44*	4.09E-02
	*UGT36D1a*	LOC109398287	2.47	8.99	3.30*	1.98E-03	8.04	2.89*	5.77E-03	10.11	3.31*	4.69E-13
	*UGT36D1b*	LOC109429550	3.51	15.58	3.66*	2.70E-03	11.07	2.54*	6.20E-04	15.75	3.32*	1.32E-18
	*UGT49B1*	LOC109410284	14.67	47.18	2.97*	3.85E-03	26.15	1.61	1.63E-01	52.21	2.90*	2.05E-26
	*UGT49C1a*	LOC109430808	1.73	14.04	7.07*	1.01E-08	6.31	3.22*	1.35E-04	12.54	5.73*	5.52E-10
	*UGT49C1b*	LOC109426260	12.43	58.15	4.56*	1.65E-02	18.99	1.37	4.81E-01	34.03	2.22*	5.97E-11
	*UGT5*	LOC109408672	3.87	17.61	4.40*	4.60E-03	10.07	2.38*	1.87E-02	16.88	3.59*	4.69E-13
ABCs	*ABCG1*	LOC109428250	16.05	46.11	2.63*	6.16E-03	22.86	1.29	5.84E-01	52.64	2.68*	3.31E-19
	*ABCG1-X1*	LOC109429973	6.41	16.74	2.34*	6.77E-03	12.10	1.69	3.13E-02	16.13	2.03*	1.26E-08
	*ABCG4*	LOC109429564	4.94	18.95	3.51*	1.07E-03	6.12	1.10	8.15E-01	17.51	2.82*	1.67E-12

*The value of Fold Change followed by the symbol * represents that the gene is significantly over-expressed in the field population relative to the susceptible strain (SS). The nomenclature of all the P450 genes of *Ae. albopictus* in this study was performed by David R. Nelson according to the traditional nomenclature rules.

## 4 Discussion

Insecticide resistance in *Ae. albopictus* is now threatening the global fight against arboviral human diseases such as dengue, yellow fever, chikungunya, and Zika (Asgarian et al., 2023). In China, insecticide resistance in *Ae. albopictus* is rapidly emerging in most areas (Li et al., 2018; Gao et al., 2018; Su et al., 2019; Yuan et al., 2023; Zhao et al., 2023). Therefore, monitoring and management of insecticide resistance is becoming a crucial element in vector control. The purpose of this study was to evaluate the insecticide resistance status and screen for genes potentially involved in resistance in *Ae. albopictus* field populations in the urban areas of Beijing.

Bioassay results reveal that the three examined populations have developed varying degrees of resistance to the commonly used pyrethroid and carbamate insecticides, and the extent of resistance is related to the intensity of insecticide usage against different *Ae. albopictus* populations (Table 1). For example, in JK, where insecticide sprays were applied three times each month from July to September during the years 2016–2019, *Ae. albopictus* developed the highest resistance to deltamethrin, permethrin, and propoxur. By contrast, no significant resistance to malathion was detected in all the three populations, probably because organophosphate insecticides have been used less frequently. The observations that these populations were susceptible to malathion, but resistant to propoxur, may suggest that propoxur resistance is attributed to enhanced detoxification by enzymes that selectively catalyze propoxur rather than mutations in their shared target acetylcholinesterase.

Similarly, *Ae. albopictus* populations in the urban areas of Guangzhou in southern China have developed resistance to deltamethrin, permethrin, propoxur, and bendiocarb, but they have remained susceptible to malathion (Li et al., 2018; Su et al., 2019); field populations of *Ae. albopictus* from Zhejiang Province in eastern China were widely resistant to pyrethroids but sensitive to organophosphate insecticides (Chang et al., 2014). A recent study also reported that seven populations from Guangyuan City of Sichuan Province in western China are susceptible to malathion but resistant to beta-cypermethrin and deltamethrin (Zhao et al., 2024). The above findings from different provinces of China suggest that most urban *Ae. albopictus* mosquitoes may be subjected to similar selective pressures of insecticides and consequently exhibit similar profiles of insecticide resistance. Although the annual most active period of *Ae. albopictus* in Beijing is relatively short (from July to August), the large-scale mosquito control campaigns in urban areas have imposed continuing pressure to select for insecticide-resistant populations of *Ae. albopictus*. The prevalence of phenotypic resistance in *Ae. albopictus* populations in Beijing strongly suggests that reduced use or rotation of insecticides should be considered in future practices of vector control and highlights the necessity to understand the genetic mechanism underlying multiple insecticide resistance.

It has been extensively accepted that enhanced metabolic detoxification of insecticides is a major mechanism in insecticide resistance in mosquitoes (Liu, 2015). To identify genes that are associated with insecticide resistance, we sequenced 12 *Ae. albopictus* transcriptomes. A total of 586 upregulated and 512 downregulated genes were commonly observed in the DK, GJK, and JK populations as compared with the SS strain (Figure 1). Insecticide resistance often incurs a metabolic cost, so downregulated genes may reflect suppression of non-essential pathways to conserve resources. KEGG pathway enrichment analysis of DEGs revealed that several detoxification genes were upregulated in the field-resistant populations. There are 186 P450, 64 CCE, 32 GST, and 71 ABC transporter genes in the *Ae. albopictus* genome (Chen et al., 2015), of which 18 P450, 5 GST, 4 CCE, 8 UGT, and 3 ABC genes were significantly expressed in the three field populations relative to the susceptible strain, indicating that detoxification-mediated mechanisms may play crucial roles in the phenotypic resistance to pyrethroids (Marcombe et al., 2024) and carbamates in *Ae. albopictus*.

P450s are well recognized for their roles in insecticide metabolism and resistance. Of the 18 P450 genes that are overexpressed in at least one of the three field populations, 9 genes are members of the CYP9J subfamily (Table 2). P450 genes belonging to the CYP9J subfamily (CYP9J6, CYP9J8, CYP9J9, CYP9J10, CYP9J19, CYP9J22, CYP9J23, CYP9J24, CYP9J26, CYP9J27, CYP9J28, CYP9J31, and CYP9J32) were also reported to be overexpressed in several pyrethroid-resistant populations of *Ae. aegypti* (Strode et al., 2008; Saavedra-Rodriguez et al., 2012; Stevenson et al., 2012; Rahman et al., 2021; Bariami et al., 2012). Moreover, functional studies *in vitro* demonstrate that some CYP9J members (e.g., CYP9J24, CYP9J26, CYP9J28, and CYP9J32) are capable of metabolizing pyrethroids permethrin or deltamethrin (Stevenson et al., 2012). Moreover, we observed two members (CYP6Z8 and CYP6Z23) of the CYP6Z subfamily over-transcribed in two (DT and JK) of the three field populations. The involvement of CYP6Z in insecticide resistance in other mosquito species was also reported. For example, CYP6Z8 and CYP6Z9 were found to play a pivotal role in pyrethroid clearance in *Ae. aegypti* (Bariami et al., 2012; Chandor-Proust et al., 2013); three CYP6Z genes in *Ae. albopictus* were involved in the resistance to deltamethrin *in vivo* (Zou et al., 2024). In addition, upregulation of CYP6AG17 was also observed in DT and JK populations in this study. Three CYP6AG (Dusfour et al., 2015; Dusfour et al., 2019; Zulfa et al., 2022) homologous genes were associated with deltamethrin resistance in field *Ae. aegypti* (Saavedra-Rodriguez et al., 2019). These observations coupled with the mRNA levels (Table 2) make us hypothesize that CYP6Z8, CYP6Z23, and CYP9J18 may be key players in P450-mediated insecticide resistance in *Ae. albopictus* urban populations in Beijing.

In this study, five glutathione S-transferases (GSTs) were observed to be overexpressed in the 3 field populations (Table 2). GSTs may contribute to the development of resistance to all main classes of insecticides via direct metabolism and/or sequestration of insecticides or via indirectly providing protection against oxidative stress induced by insecticide exposure (Pavlidi et al., 2018; Zhai et al., 2024). GSTs were found to be related with pyrethroid resistance in other species of mosquitoes such as *Ae. aegypti* (Strode et al., 2008; Lumjuan et al., 2011; Seixas et al., 2017; Leong et al., 2019), *An. sinensis* (Zhu et al., 2014), and *An. gambiae* (Bonizzoni et al., 2015). GST activities were enhanced in most *Ae. albopictus* populations from Sri Lanka resistant to pyrethroids (NWNP et al., 2021). Notably, the abundances of GSTe2a and GSTI1 in the three field populations were significantly higher than those of the susceptible strain (Table 4), indicating that these two GSTs may play crucial roles in resistance.

The CCE family contains both catalytically active enzymes and non-catalytic proteins and can act by rapid binding or sequestration to affect the interactions between insecticides and targets (Mourya et al., 1993; Karunaratne et al., 1995; Safi et al., 2019). In the current study, four CCEs were observed to be overexpressed in at least one of the three field populations (Table 2). Notably, COesterase6a had the highest abundance and was commonly overexpressed in all the three resistant populations, highlighting its importance in resistance. Interestingly, the homologous COesterase5a and COesterase6a genes were reported to undergo an independent amplification respectively and spread between two countries in field-resistant *Ae. albopictus* populations (Grigoraki et al., 2017).

In addition to the above-mentioned detoxification enzymes, an increasing number of studies have indicated the involvement of UGTs and ABC transporters in insecticide resistance (Bariami et al., 2012; Dermauw and Van Leeuwen, 2014; Lv et al., 2016; He et al., 2019; Rault et al., 2019; Zhou et al., 2019b; Bruckmueller and Cascorbi, 2021; Israni et al., 2022; Chen et al., 2023). In this study, we found that 8 UGTs and 3 ABCs were upregulated in the three resistant populations relative to the susceptible strain of *Ae. albopictus* (Table 2). Further research is required to clarify the role of these DEGs in resistance.

This study has some limitations. First, the diagnostic dosage method could only determine whether the field population are resistant or susceptible to the insecticides based on survival rates at the discriminating dose, without quantifying the resistance levels or detecting low levels of resistance or early-stage resistance development. Second, the differences in some genes expressions observed between the susceptible strain and field populations could be due to the differences in the genetic background and/or living (rearing) conditions rather than insecticide resistance *per se*. Including a field-susceptible strain would help address these potential confounding factors and provide a more robust analysis of the genetic basis of insecticide resistance in *Ae. albopictus*. This approach would provide a more accurate baseline for comparison. However, to our knowledge, it is quite difficult to find a susceptible strain sourced from the field due to the massive insecticide use historically. Another limitation of the study was the lack of the supporting biochemical data or synergist such as piperonyl butoxide (PBO) and S, S, S-tributyl phosphorothioate (DEF) assay. However, our research group is currently conducting *in vivo* and *in vitro* functional validations on three overexpressed P450 genes, aiming to clarify their contribution to insecticide tolerance.

In summary, the availability of *Ae. albopictus* genomes (Chen et al., 2015; Palatini et al., 2020) has greatly benefited transcriptome analyses. The high-quality transcriptomes obtained in the study allow us to make a list of potential genes involved in metabolic resistance to insecticides in the field-resistant populations. Our results support the notion that the three major detoxification enzyme families (P450s, CCEs, and GSTs) together with ABCs and UGTs are involved in insecticide resistance in *Aedes* mosquitoes (Kasai et al., 2014; Chen et al., 2015; Strode et al., 2008; Zhu et al., 2014; Lv et al., 2016; Liu et al., 2011; Simma et al., 2019; Lien et al., 2019; Messenger et al., 2021). The exact contribution of the above-mentioned detoxification-related genes to resistance will require further work. Such effort is underway in our lab.

## 5 Conclusion

Bioassay results demonstrate that all the examined *Ae. albopictus* field populations in Beijing have developed multiple phenotypic resistance to commonly used pyrethroids and carbamate. The identification of several DEGs associated with insecticide resistance indicates that the mechanisms underlying resistance in field populations are complicated. The observation that multiple detoxification-related genes are upregulated in all the three resistance populations strongly suggests that enhanced detoxification mediated by increased expression of detoxifying enzymes may play important roles in the development of multiple resistance. The significant resistance status detected in all the three populations highlights that resistance management strategies such as insecticide rotation and/or non-chemical-based measures should be implemented in order to sustain effective control of the disease vector and vector-borne diseases.

## Data Availability

All the reads generated in transcriptome sequencing were deposited in the NCBI Sequence Read Archive (SRA) database with BioProject accession number PRJNA1110376.
