# Supplementary material for: Insecticide resistance in the field populations of the Asian tiger mosquito Aedes albopictus in Beijing: resistance status and associated detoxification genes
Source: Front Physiol. 2024 Dec 18;15:1498313. doi: 10.3389/fphys.2024.1498313 (PMC11688286; doi:10.3389/fphys.2024.1498313)
Supplement: Supplementary file 2 [file Table1.docx]

Supplementary file 1. Quality control of transcriptome sequencing data of 12 *Ae. albopictus* samples

| **Samples** | **Raw reads** | **Raw bases** | **Clean reads** | **Clean bases** | **Error rate (%)** | **Q20 (%)** | **Q30 (%)** | **GC content (%)** |
| --- | --- | --- | --- | --- | --- | --- | --- | --- |
| **DT1** | 50191200 | 7578871200 | 49853362 | 7430700556 | 0.0245 | 98.26 | 94.59 | 47.55 |
| **DT2** | 50389952 | 7608882752 | 50060010 | 7460487139 | 0.0241 | 98.43 | 95.06 | 49.83 |
| **DT3** | 60226550 | 9094209050 | 59771616 | 8905342614 | 0.0246 | 98.22 | 94.52 | 49.72 |
| **GJK1** | 54851868 | 8282632068 | 54480712 | 8130537387 | 0.0242 | 98.36 | 94.88 | 51.1 |
| **GJK2** | 52569696 | 7938024096 | 52169710 | 7765345158 | 0.0253 | 97.95 | 93.84 | 47.65 |
| **GJK3** | 56106992 | 8472155792 | 55728090 | 8309890902 | 0.0246 | 98.23 | 94.48 | 46.86 |
| **JK1** | 53127720 | 8022285720 | 52761254 | 7878827988 | 0.0248 | 98.15 | 94.35 | 50.27 |
| **JK2** | 51615850 | 7793993350 | 51250646 | 7647057622 | 0.0248 | 98.15 | 94.33 | 50.04 |
| **JK3** | 51381180 | 7758558180 | 50996580 | 7607666011 | 0.0249 | 98.11 | 94.26 | 50.3 |
| **SS1** | 47513602 | 7174553902 | 47197650 | 7047621846 | 0.0242 | 98.36 | 94.91 | 50.51 |
| **SS2** | 45768000 | 6910968000 | 45448318 | 6795273193 | 0.0243 | 98.35 | 94.86 | 50.59 |
| **SS3** | 49174032 | 7425278832 | 48841460 | 7287840077 | 0.0242 | 98.37 | 94.92 | 50.29 |
