# Supplementary material for: Insecticide resistance in the field populations of the Asian tiger mosquito Aedes albopictus in Beijing: resistance status and associated detoxification genes
Source: Front Physiol. 2024 Dec 18;15:1498313. doi: 10.3389/fphys.2024.1498313 (PMC11688286; doi:10.3389/fphys.2024.1498313)
Supplement: Supplementary file 3 [file Table2.docx]

Supplementary file 2. Clean reads mapped to reference genome of each sample

| **Samples** | **Clean reads** | **Total mapped** | **Multiple mapped** | **Unique mapped** |
| --- | --- | --- | --- | --- |
| DT1 | 49853362 | 41442538(83.13%) | 10401490(20.86%) | 31041048(62.26%) |
| DT2 | 50060010 | 42013586(83.93%) | 9234575(18.45%) | 32779011(65.48%) |
| DT3 | 59771616 | 50163669(83.93%) | 11191987(18.72%) | 38971682(65.2%) |
| GJK1 | 54480712 | 46264958(84.92%) | 10142249(18.62%) | 36122709(66.3%) |
| GJK2 | 52169710 | 43581000(83.54%) | 10837523(20.77%) | 32743477(62.76%) |
| GJK3 | 55728090 | 46661425(83.73%) | 12013534(21.56%) | 34647891(62.17%) |
| JK1 | 52761254 | 44149011(83.68%) | 9384150(17.79%) | 34764861(65.89%) |
| JK2 | 51250646 | 42664925(83.25%) | 8929881(17.42%) | 33735044(65.82%) |
| JK3 | 50996580 | 42469011(83.28%) | 8695833(17.05%) | 33773178(66.23%) |
| SS1 | 47197650 | 39603801(83.91%) | 8097232(17.16%) | 31506569(66.75%) |
| SS2 | 45448318 | 38207321(84.07%) | 7632716(16.79%) | 30574605(67.27%) |
| SS3 | 48841460 | 41545101(85.06%) | 9135671(18.7%) | 32409430(66.36%) |

Note: Multiple mapped: indicates that the same read can be matched to ≥2 genes, which is often due to the presence of paralogous homologs with high similarity in the genome, or gene duplication. Unique mapped: indicates that the same read can only be compared to 1 gene.
